# IPM thresholds for *Agriotes* wireworm species in maize in Southern Europe

**DOI:** 10.1007/s10340-014-0583-5

**Published:** 2014-04-08

**Authors:** Lorenzo Furlan

**Affiliations:** Veneto Agricoltura, Agripolis, via dell’Università 14, Legnaro, PD Italy

**Keywords:** Wireworms, *A. brevis*, *A. sordidus*, *A. ustulatus*, IPM, Bait traps

## Abstract

Currently, integrated pest management (IPM) of wireworms is not widespread in Europe. Therefore, to estimate the densities of three major wireworm species in southern Europe (*Agriotes brevis* Candeze, *A. sordidus* Illiger, and *A. ustulatus* Schäller), bait traps were deployed pre-seeding in maize fields in north-eastern Italy between 1993 and 2011. Research discovered that there was a significant correlation between all three wireworm species caught in the bait traps and damage to maize plants, but damage symptoms varied. Wherever *A. ustulatus* was the main species caught, there was no significant damage to maize plants, but seeds were damaged. Most of the symptoms caused by *A. brevis* and *A. sordidus* were to the central leaf/leaves, which wilted because of feeding on the collar. *A. brevis* was the most harmful species; when more than one *A. brevis* wireworm was caught per trap, plant damage sometimes resulted in reduced yield. Five *A. ustulatus* larvae per trap caused the same damage to maize as one *A. brevis*. *A. sordidus* came second (threshold two larvae/trap). These thresholds are reliable for: (1) bare soil in which there are no alternative food sources; (2) average soil temperature 10 cm beneath the surface of above 8 °C for 10 days; (3) soil humidity near to field water capacity, but days of flooding have not been considered. The implementation of the practical method described herein may lead to effective IPM of wireworms in maize and to a significant reduction in the number of fields treated with soil insecticides.

## Introduction

EU Directive 2009/128/EC on the sustainable use of pesticides makes it compulsory to implement integrated pest management (IPM) for annual crops in Europe from January 2014. IPM strategies have not played a significant role in these crops to date, yet they have been widely used for crops such as orchards and vineyards. Therefore, accurate information about IPM strategies for annual crops is needed urgently, but this information must take into account that arable farming has few resources in terms of income, labour and technology. Since the use of soil insecticides is widespread, this paper intends to provide reliable IPM information to tackle wireworms, the main soil pest in Europe (Furlan 2005). It has proved difficult to implement IPM strategies for wireworms in Europe due to a shortage of reliable information on how to assess population levels and the relative thresholds (Furlan 2005). Wireworms are the larvae of click beetles (Coleoptera: Elateridae), but damage-causing genera and species vary with geographic location (Furlan et al. 2000, 2001b, 2007a; Rusek 1972; Staudacher et al. 2013). In Europe, most larvae in agricultural land belong to the *Agriotes* genus, but the specific species must be established if we are to predict the potential damage to crops. For example, high populations of *Agriotes ustulatus* do not damage maize late in the spring (late May–June) because most of the larvae are in a non-feeding phase (Furlan 1998); in the same period, however, *Agriotes sordidus* or *A. brevis* can seriously reduce the stand of maize crops (Furlan 2004). The adults (click beetles) of these species can be divided into two main groups: (i) adults that do not overwinter and lay eggs a few days after swarming (*A. ustulatus* Schäller and *A. litigiosus* Rossi); and (ii) adults that overwinter, live for months, and lay eggs for a long period after adult hardening (*A. sordidus* Illiger, *A. brevis* Candeze, *A. lineatus* L., *A. sputator* L., *A. obscurus* L., *A. rufipalpis* Brullè, and *A. proximus* Schwarz) (Furlan 2005). The life cycle of the species in both groups is about 24–36 months. In spring, the larvae of group (i) entering the bait traps come from eggs laid two years before, but group (ii) larvae come mainly from eggs laid the previous year. Unfortunately, the vast majority of literature on this matter does not report which species were involved (Hinkin 1976; Chabert and Blot 1992). Therefore, this present research assesses the effect of various *Agriotes* species on maize and looks at thresholds based on wireworms caught in bait traps in order to establish a range of IPM strategies. The ultimate aim of the research is to provide practical information so that European farmers can implement reliable, feasible and affordable IPM strategies to prevent wireworms damaging their maize.

## Materials and methods

### Field sites

Research was conducted in north-east Italy (area covered: 45.64N, 12.96E and 45.05N 11.88E) from 1993 to 2011 (19 consecutive years) on fields with the following characteristics: (1) soil at field water capacity, i.e. no more water can be stably retained; after winter, all of the fields studied, and particularly the bare ones, i.e. no crops consuming water, are usually very humid due to rainfall, negligible evaporation and transpiration. Sometimes strong winds dried up the soil, but only the top-most layer and not where the traps were placed. Therefore, the soil layer containing the traps was always at field water capacity; (2) bare soil (no plants growing), since traps perform reliably when they do not have to compete with plants whose roots produce carbon dioxide, which attracts larvae (Doane et al. 1975); (3) several previous crops had been sown, such as maize, soybean, winter cereals and meadow (e.g. alfalfa, festuca); meadow must be ploughed at least three months before the bait traps are placed in order to make sure that all ploughed-up meadow plants have died (it was observed that this takes about three months); the main reason for this procedure is that it allows the bait traps to attract wireworms without the competition of plants, as described above. Each year, monitoring was conducted in fields representing a balanced sample of agronomic conditions in north-east Italy. Part of the soils was classified with the USDA soil texture triangle based on analyses carried out in accordance with official USDA methods. Soil pH was basic for all the fields and ranged between 7.9 and 8.3.

### Agronomic practices

Conventional agronomic practices were applied to all of the fields studied (i.e. ploughing, harrowing, fertilization with 240–300 N kg, 70,000–76,000 seeds/ha, interrow width 75 cm, pre-emergence plus post-emergence herbicide treatments causing very low weed densities, and planting date from late March to late April). The following commercial hybrids were used: ANITA, COSTANZA, ALICIA, SENEGAL (1993–2001); TEVERE (2002–2004); DKC6530 (2005–2006); DKC 6530, MITIC, KERMESS, KLAXON (2007–2008); DKC6666, NK FAMOSO, PR31A34, PR32G44 (2009–2010); and DKC6677, PR32G44 and NK FAMOSO (2011).

### Estimation of wireworm population level

Bait traps made and used in accordance with Chabert and Blot (1992) were deployed to estimate wireworm population densities from late February to mid April. These and similar traps were found to be efficient at capturing wireworms after research in UK conditions (Parker 1994, 1996). Each trap comprised a plastic pot 10 cm in diameter with holes in the bottom. The pots were filled with vermiculite, 30 ml of wheat seeds and 30 ml of maize seeds; they were then moistened before being placed into the soil 4–5 cm below the soil surface, after which they were covered with an 18-cm diameter plastic lid placed 1–2 cm above the pot rim. Traps were hand-sorted after 10 days when the average temperature 10 cm beneath the surface was above 8 °C (Furlan 1998, 2004) to ensure that the bait traps stayed in the soil for an equal period of wireworm activity. *Agriotes* larvae do not feed, or feed very little, at lower temperatures. Generally, the traps were removed from the fields 2 to 8 days before maize seeding. No considerable differences in wireworm feeding activity were observed between 8 and 13 °C, which is the usual temperature range in early spring in northern Italy (Furlan 1998, 2004). Previous investigations (unpublished data) found that only a negligible number of larvae escaped from the traps since it was noted that numbers tended to increase as days passed (Furlan personal observation). The final number of larvae was assessed under the aforementioned conditions, regardless of larvae behaviour on individual days. Population levels were calculated only on days when humidity was close to field water capacity. Dry top-soil forces larvae to burrow deep beneath the surface, away from the bait traps (Furlan 1998), and high humidity (flooding in extreme cases) prevents larvae activity since all the soil pores are full of water and contain no oxygen. Therefore, any days on which these conditions occurred were excluded from calculations, regardless of the soil temperature. This obviously resulted in traps sometimes being kept in the soil for longer than 10 days. In the UK, Parker (1994) caught large numbers of *Agriotes* wireworms in average soil temperatures that ranged from 5 to 10 °C. In order to recover as many larvae as possible, and thus increase research precision, after 10 or more days, the traps were inspected manually and the contents put into Berlese funnels fitted with a 0.5-cm mesh screen at the bottom. The trap contents were allowed to dry for at least 20 days in a sheltered place without lamps, and the larvae that fell into the collecting vials were counted and identified. A personal key (unpublished), developed by rearing single larvae to adults, was used to identify them. Some of the distinguishing characteristics complied with Rudolph (1974). Adults were determined with the key in Platia (1994). The traps were deployed on a grid (20 m × 10 m apart); a minimum of nine bait traps was placed per field and the sample area varied between 0.2 and 1 ha. The larger the area to be covered, the higher the number of traps placed. A total of 5,400 traps were placed during this 19-year study (18 traps/field on average). This research encompassed only fields monitored in spring (early March to late April).

### Estimation of wireworm damage to maize

In the maize fields monitored, wireworm damage to seeds and plants was assessed only once it was sure that insecticides had not been used, or that random untreated maize strips/plots, 3 or 4.5 m wide, had been sown alternately with treated strips/plots. When strips/plots were treated, the most effective insecticides available were used: 1993–1994: Diphonate® (Fonofos 4.75 % a.i.) 10 kg/ha of granules applied in-furrow; Dotan® (Chlormephos 4.95 % a.i.) 7 kg/ha of granules applied in-furrow; 1995–2005: Fipronil (Regent TS®) 0.6 mg a.i./seed; Imidacloprid (Gaucho®) 1.2 mg a.i./seed; Regent 2G® (Fipronil 2 % a.i.) 5 kg/ha of granules applied in-furrow; 2006–2010: Force® ST (Tefluthrin 0.5 % a.i.) 15 kg/ha of granules applied in-furrow; Clothiadinin (Poncho®) 0.5 mg a.i./seed; 2011: Force® ST (Tefluthrin 0.5 % a.i.) 15 kg/ha of granules applied in-furrow; Clothiadinin (Poncho®) 0.5 mg a.i./seed; Imidacloprid (Gaucho®) 1.2 mg a.i./seed.

One litre of the fungicide Metalaxil + Fludioxonil (Celest®) per tonne of seed was used to treat all of the maize seeds planted. In order to study the correlation between wireworm densities (larvae/bait trap) and the damage to maize, at the 2–3 and 6–8 leaf stages, two sub-plots of 4 × 20 m rows of maize per portion of untreated field (0.1–0.2 ha) or untreated strip were chosen at random and the plants observed. During plot trials, all plants (healthy and damaged) at the centre of each untreated plot were counted; the plots covered an area of 15–18 m × 1.5 m. The location and the number of the sub-plots were the same in both the untreated/treated strips and completely untreated field. In order to assess wireworm damage on emerged plants, plants with typical symptoms (e.g. wilting central leaves, broken central leaf due to holes in the collar, wilting of whole small plants) were sought and the soil around the plants was dug up to a depth 5–6 cm; any larvae found near the collar were collected and identified. Wherever maize plants were missing from the rows, the soil was dug up in order to assess possible wireworm damage to seeds and/or emerging seedlings. Total plant damage was calculated as the sum of damage to emerged plants and seeds. In order to establish the effect of wireworm damage on yield, the same observations were made on the treated strips/plots where used. Finally, the strips and the plots were harvested and the maize grain weighed. Maize grain samples were collected and their humidity measured with a Pfeuffer-Granomat (the same machine was used for all samples each year). The four fields in which maize stands were irregular and damaged due to factors other than wireworm activity (e.g. bird damage, low emergence due to low soil moisture, flooding) were not considered. In order to isolate the “wireworm damage effect”, analysis excluded the two fields under considerable pressure from factors other than wireworms (e.g. other parasites, viruses). Fields in which the general conditions were good, but the soil insecticide had not worked completely and the stand of treated maize plots was not optimal, were not used to evaluate the effect on yield (this happened in two cases only). In the remaining fields where the insecticides had worked effectively, the final stand of the treated strips/plots was suitable for assessing whether yield had been reduced (>90 % of the sown seeds).

### Statistical methods

All analyses were performed by SAS 9.3 (SAS Institute Inc., Cary, NC). Linear regression analysis was used to determine the relationship between damage to maize (total plant damage, emerged plant damage and seed damage) and pre-seeding catches of wireworms in bait traps for each species. A paired *t* test was used to assess the effect of wireworm damage on grain yields in treated and non-treated plots. Where soil characteristics were available, a generalized linear model (Nelder and Wedderburn 1972) with a Poisson distribution was used to determine the factors affecting the percentage of total damage for each species. The model included the effect of the main agronomic characteristics (soil texture as a fixed effect, plus organic matter content and pH as covariates) and captures/plant damage data (as covariates too). The soil types (levels of the variable) were classified as follows: clay, loam, clay loam, silt clay loam, loam, sandy loam, and loamy sand. Analysis produced least squares means estimates of parameters and risk ratio. Risk ratio measures relative effect expressed by the outcome in two groups, i.e. the ratio between the prevalence in the exposed group (numerator level) vs the non-exposed group (denominator or reference value). The type of soil with the highest damage level caused by each species was chosen as reference value. Analysis was performed with PROC GENMOD.

## Results

### Species composition and factors affecting the level of damage

Wireworms were found in the bait traps in 206 fields (70 %). The main species found were *A. brevis, A. sordidus* and *A. ustulatus*. All of these species are widespread in central and southern Europe (Furlan 1996, 2004; Furlan et al. 2000, 2007a; Kausnitzer 1994) including areas with significantly different conditions from those of this study, e.g. in Austria, *A. brevis* were found in zones with acid pH (Staudacher et al. 2013). The presence of other Elateridae species (mainly *Synaptus filiformis* Fabricius, *Melanotus* spp., *Adrastus rachifer* Geoffroy in Fourcroy) was negligible. Bait traps caught a single species in 81.1 % of the fields. The combinations of different species observed in the other cases are described in Table 1. Only four fields (1.9 %) had a considerably mixed population (two or three species in a single bait trap). Table 2 covers the fields in which at least one trap caught wireworms and gives the average, standard deviation and maximum value of all the single averages, standard deviations and maximum numbers estimated in each of the fields monitored. The variability between bait traps was high, and the ratio between average mean and average standard deviations was one. The generalized linear model found that the percentage of total damage variability was mainly explained by wireworm density (the average number of larvae/bait trap) for all three of the species studied (Table 3, *P* < 0.001). Soil texture affected the risk: loam soils were prone to higher damage risk by *A. sordidus*, while the risk of damage by *A.* *ustulatus* was much lower in clay soils. PH variations in the range of soils studied (mean = 8.01, SD = 0.11) did not influence the risk of damage by any of the species, but organic matter content (mean = 1.93, SD = 0.49) may vary the risk of damage by *A.* *ustulatus* (Table 3, *P* < 0.001).

**Table 1:** Wireworms found in the fields monitored with bait traps pre-seeding; fields are divided in accordance with the number of species found in each one

Species	Fields					
	Total	One species	*Agriotes brevis* + *Agriotes sordidus*	*Agriotes brevis* + *Agriotes ustulatus*	*Agriotes* *sordidus* + *Agriotes* *ustulatus*	All three species
Fields (no.)	206	167	9	8	12	10
*A. brevis* larvae	2,431	1,959	89	197	0	186
*A. sordidus* larvae	1,486	1,353	85	0	30	18
*A. ustulatus* larvae	4,217	3,765	0	160	280	12

**Table 2:** Variability between the number of wireworms in the single bait traps placed in fields monitored

	*Agriotes brevis*			*Agriotes sordidus*			*Agriotes ustulatus*		
	Mean	SD	Max	Mean	SD	Max	Mean	SD	Max
Mean	0.61	0.41	1.50	0.38	0.49	1.60	1.04	1.13	4.11
SD	3.27	1.71	6.43	0.65	0.61	2.05	3.25	2.88	10.58
Max	24.88	14.18	53	3.58	2.99	9	21.80	17.51	60

Average, standard deviation (SD) and maximum value of the all averages, standard deviations and maximum numbers calculated per each of the fields monitored. Only fields with average higher than zero have been considered (*Agriotes brevis* 48 fields, *Agriotes sordidus* 103 fields, *Agriotes ustulatus* 55 fields)

**Table 3:** Least squares means (% of total damage on plants) and risk ratio for *Agriotes ustulatus*, *Agriotes sordidus* and *Agriotes* *brevis* in different soils and different pH levels, percentage of organic matter and number of larvae/trap calculated with a generalized linear model

Variable	Number of fields	Least squares means % Damage (SE)	RR (95 % CI)	Chi-square	*P*
Agriotes ustulatus					
Soil				25.96	<0.001
Clay	7	0.27 (0.12)	0.22 (0.19–0.25)	12.86	<0.001
Loam^a^	31	1.24(0.23)			
Clay loam	3	0.08 (0.13)	0.06 (0.002–1.78)	2.62	0.105
pH			–	–	–
Organic matter (%)			2.04 (1.23–3.39)	7.53	<0.001
No. larvae/trap			1.25 (1.21–1.28)	455.42	<0.001
Agriotes sordidus					
Soil				67.50	<0.001
Silty clay loam	2	1.37 (1.08)	0.35 (0.07–1.67)	1.74	0.187
Loam	9	1.30 (0.44)	0.33 (0.16–0.67)	9.29	0.002
Clay loam	15	0.63 (0.19)	0.16 (0.09–0.29)	36.05	<0.001
Silty clay loam	12	2.18 (0.63)	0.55 (28–1.06)	3.16	0.076
Sandy loam	9	2.29 (0.52)	0.58 (0.36–0.93)	5.07	0.024
Loamy sand^a^	32	3.96 (0.46)			
pH			0.24 (0.03–2.22)	1.50	0.221
Organic matter (%)			0.59 (0.25–1.37)	1.74	0.188
No. larvae/trap			1.96 (1.74–2.21)	106.19	<0.001
Agriotes brevis					
Soil					
Clay	11	11.73 (1.19)	0.55 (0.32–0.94)	4.78	0.029
Loam	4	2.84 (1.13)	0.13 (0.04–0.40)	12.69	<0.001
Clay loam	8	8.45 (1.26)	0.40 (0.23–0.67)	11.96	<0.001
Loamy sand^a^	2	21.36 (5.58)			
pH			15.40 (0.29–>20)	1.82	0.178
Organic matter (%)			1.27 (0.56–2.88)	0.32	0.569
No. larvae/trap			1.07 (1.06–1.09)	128.49	<0.001

*RR* risk ratio*, SE* standard error, *CI* confidence interval

^a^Represents the reference level of comparison in the calculation of risk ratio

### The correlation between species caught by bait traps and symptoms observed on maize plants

**Fig. 1:**
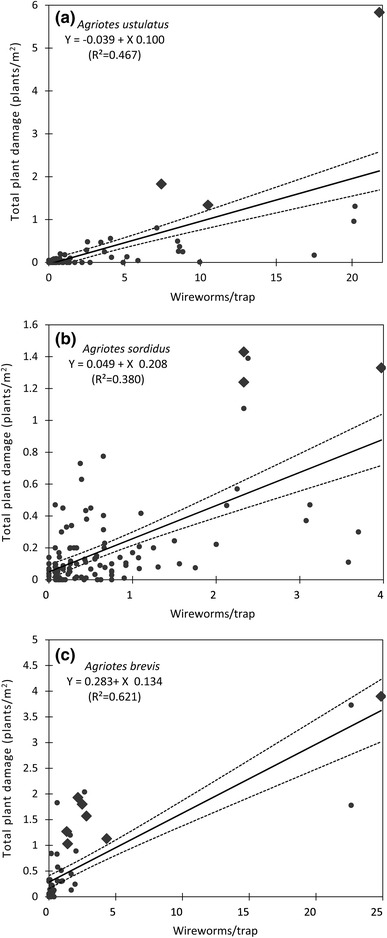
The relationship between wireworm density (number of wireworms/bait trap) and total plant damage (plants/m²) for *Agriotes ustulatus*, *A. brevis* and *A. sordidus* (±95 % average confidence level). *Larger* (rhomb) *dots* represent combinations that resulted in a significant yield reduction

Symptoms on maize plants varied per wireworm species. Wherever *A. ustulatus* was the prevalent species, no significant symptoms were found on emerged maize plants (e.g. wilting central leaves); see Table 5 and Fig. 1. Symptoms on emerged plants were always caused by *A. brevis* and/or *A. sordidus* (Table 2; Fig. 1). Only one case of serious seed damage by *A. brevis* was observed; the maize had been sown late and the seeds germinated in May due to a prolonged dry spell. No significant seed damage by *A. sordidus* larvae was observed. *A. ustulatus* larvae (Table 5) significantly affected plant stand by damaging seeds, which could not germinate or emerge when the population was high. Few maize plants had the central leaf broken by *A. ustulatus* feeding below ground; in the fields where *A. ustulatus* was the prevalent species, less than 0.1 % of plants were damaged (3 out of a total of 3,100 seeds + plants found damaged). Broken central leaves were restricted to the 3–4 leaf stage. *A. brevis* and *A. sordidus* proved able to cause all of the possible symptoms and to damage even developed maize plants (up to the 8–10 leaf stage). Most of the damaged plants had one or more wilted central leaves due to larval feeding on the collar, which sometimes killed them.

### The correlation between species caught by bait traps and damage to maize

All or most of the larvae collected from damaged seeds, seedlings or plants belonged to the prevalent species captured by the bait traps (Table 4). A significant correlation (for all species) was discovered between the average number of wireworms caught in bait traps and the total damage to maize (damage to seeds, plus damage to emerged plants; Table 5, Fig. 1). *A. brevis* was the most harmful species, as even wireworm densities just over one wireworm/trap caused considerable plant damage (one to two plants attacked/m²), i.e. enough to reduce yield (Fig. 1; Table 6). The graph shows either a very low or a high population (only three fields had a very high population) and almost nothing in between. During this 19-year research, high *A. brevis* populations were found in maize fields after meadow had been ploughed, or after a soil had been continuously covered with vegetation (e.g. soybeans just after winter-wheat in the same growing season). After the first year of maize, the wireworm populations decreased dramatically; this means that high populations are possible, but uncommon, as they occurred only in a few meadows and fields where crops were continuously planted. Low populations, however, were common, as levels fell the very next spring, and usually remained low for several years after. Intermediate populations are therefore rare. To cause the same level of damage in maize fields, five times more *A. ustulatus* larvae are needed (Fig. 1; Table 6). In Fig. 1, the notable outlier in the *A. ustulatus* graph concerns a 2010 trial; the results may be explained by a cold spring and a very compact soil, which significantly slowed the emergence of maize seedlings, leaving them in the soil for a long time (about 20 days). These soil and climatic conditions did not cause significant damage in other fields with lower wireworm populations. *A.* *ustulatus* caused almost identical total damage and seed damage because it harmed very few emerged plants; on the contrary, very few maize seeds were damaged by *A.* *brevis* and *A. sordidus*. *A. sordidus* was the second most harmful with wireworm densities above two larvae/trap leading to reduced yield (Fig. 1; Table 6). In Fig. 1 (*A. sordidus*), the outlier fields, which experienced a significant decrease in yield, had sandy loam soils. Similar population levels did not cause serious damage in heavy soils. In most fields (0–1 larva/trap), wireworm damage was negligible and did not cause any visible effects on maize crops, i.e. less than 5 % of plants were attacked and, in most cases, they partially or completely recovered. In some cases, damage of over 1 plant/m² led to significant yield reduction (Fig. 1). Nevertheless, in others, even very severe plant damage (>3 plants/m²; >40 %) did not result in reduced yield. For example, in the same year (2011), severe plant damage (>3 plants/m²; >50 %) resulted in significant yield reduction at one site, but another trial produced no difference between untreated plots (8.74 t/ha) and Imidacloprid-treated plots (8.59 t/ha), despite the treated plots giving much higher stands than untreated ones in both trials. Plant damage below 1 plant/m² never resulted in significant yield reduction, and there were very limited differences (ranging between 0.01 and 0.3 t/ha) between treated and untreated strips or plots (see Furlan et al. 2002, 2007, 2009a, b, 2011). The 2011 study confirmed the previous long-term observations (Table 7). A further field infested by *A. brevis* (damage >3 plants/m²; >50 %) experienced a significant yield reduction of 4.2 t/ha. The hybrid was PR32G44. Wherever wireworm densities of *A. ustulatus* were lower than five larvae/trap and *A. sordidus* were lower than two larvae/trap (Fig. 1), stand reduction was lower than 0.5 plants/m² (in most cases, less than 5 % of total plants); no fields experienced reduced yield (i.e. there were no significant differences between treated and untreated strips/plot (Table 6; Fig. 1).

**Table 4:** Wireworm species identified as damaging maize seeds and plants in fields monitored with bait traps expressed as a percentage of the total number of larvae collected from damaged plants

Fields (no.)	Species in bait traps	*Agriotes ustulatus*	*Agriotes brevis*	*Agriotes sordidus*	Others	Total number of larvae
30	*Agriotes ustulatus*	99.5	0.2	0.2	0.1	1,015
31	*Agriotes brevis*	0.1	99.6	0.2	0.1	754
88	*Agriotes sordidus*	0.1	0.2	99.7	0.0	622

This table considers only fields where bait traps caught larvae belonging to one species

**Table 5:** Statistical outputs of the linear relationships between damage to maize and pre-seeding catches of wireworms (*Agriotes* *brevis*, *Agriotes sordidus*, *Agriotes ustulatus*) in bait traps

Fields (no.)	Species	Total plant damage (plants/m²)		Seed damage (n/m²)		Emerged plant damage (plants/m²)	
		R²	*P*	R²	*P*	R²	*P*
69	*Agriotes brevis*	0.621	<0.0001	0.002	0.709	0.610	<0.0001
135	*Agriotes sordidus*	0.380	<0.0001	Not found	Not found	0.380	<0.0001
93	*Agriotes ustulatus*	0.467	<0.0001	0.469	<0.0001	0.011	0.326

“Total plant damage” is number of missing plants due to wireworm feeding on seeds (seed damage) + number of emerged plants damaged by wireworms (e.g. wilting of central leaves due to feeding on plant collars, broken central leaves)

**Table 6:** Percentage of fields where significant yield reductions occurred at different densities of the *Agriotes* wireworm species being studied (the average numbers of wireworms/trap were considered)

Wireworm species	Wireworm catches (larvae/trap)	Fields sampled (no.)	Fields with yield reduction (no.)	Fields with yield reduction (%)
*Agriotes ustulatus*	0	38	0	0.0
	0.1–1	25	0	0.0
	1.01–2	7	0	0.0
	2.01–5	9	0	0.0
	**5.01–10**	9	1	11.1
	**>10.01**	**5**	**2**	**40.0**
*Agriotes brevis*	0	21	0	0
	0.1–1	32	0	0.0
	**1.01–2**	**6**	**2**	**33.3**
	**2.01–5**	**7**	**4**	**57.1**
	**>5.01**	**3**	**1**	**33.3**
*Agriotes sordidus*	0	32	0	0.0
	0.1–1	83	0	0.0
	1.01–2	10	0	0.0
	**>2.01**	**10**	**3**	**30.0**

Bold values indicate the population levels that resulted in yield reduction

**Table 7:** Maize grain yield (t/ha of grain at 14 % humidity) in a random subset of fields with <5 % (0.2 plants/m²) wireworm (*A. sordidus* Illiger) damage in untreated and treated plots with two different maize hybrids in 2011

Treatments/hybrids	*KORIMBOS*	*DKC6677*
Untreated	11.19	13.40
*Tefluthrin*	11.34	N/A
*Clothiadinin*	N/A	13.49
Df/t/P	27/-0.550/0.587	21/-0.330/0.744

*N/A* unavailable data, *Df/t/P* degrees of freedom, *t*-value, *P*-value

## Discussion

This long-term research found a significant correlation between the number of wireworms caught in bait traps before seeding and damage to maize plants caused by three of Europe’s main wireworm species: *A. brevis, A. sordidus* and *A. ustulatus*. Over the last 19 years, whatever the hybrid, and regardless of agronomic and climatic conditions, no yield reduction was observed when *A. brevis* populations were lower than one larva/trap, *A. sordidus* populations were lower than two larvae/trap and *A. ustulatus* populations were lower than five larvae/trap. These should be considered reliable thresholds for each species. Populations were assessed via the deployment of at least nine bait traps in a sample soil grid (20 m × 10 m). Although statistical analyses show that much of the variability in wireworm plant damage cannot be explained by the wireworm densities estimated by the bait traps, i.e. high wireworm density does not always mean high damage, this study did demonstrate that serious plant damage resulting in yield reduction may only occur when wireworm populations are above the thresholds established above, provided that precise conditions occur.

### Conditions needed to use the thresholds

In order to use the thresholds established, the following conditions have to be satisfied: (i) no alternative food sources are available, soil is bare, and if meadow (e.g. alfalfa, festuca) has been cultivated previously, the field must have been ploughed at least three months before the bait traps are placed (no other previously grown crops have any particular requirements); (ii) average soil temperature 10 cm beneath the surface is above 8 °C for 10 days (including non-consecutive days); soil humidity is near to field water capacity, but days when soil humidity is over water capacity (soil pores filled with water, i.e. flooding) are not to be considered, regardless of soil temperature, since the wireworms are not active. These can be considered reliable, prudent economic thresholds for the implementation of IPM in maize in Italy and probably in the countries where the studied species are present in similar agronomic and climatic conditions. When trap catches are below the established thresholds, the probability of economic damage is negligible. However, although significant yield reduction is a risk when thresholds are exceeded, it may not always occur, as a combination of climatic and agronomic factors (e.g. hybrid, soil, rainfall, fertilization, irrigation) may compensate for stand reduction. In most cases, yield did not fall. Several factors may influence trap catches, including: (i) alternative food sources (Parker 1996); (ii) soil temperature (Furlan 1998, 2004; Chabert and Blot 1992); and (iii) soil moisture usually suitable for wireworm activity in spring in Italy and many other European countries. Thresholds, however, do need to be evaluated for different species and, for the species considered in this manuscript, under other conditions.

### Practical implementation of thresholds

Thresholds expressed as the number of wireworms per m², or per trap, that do not specify the species caught (e.g. Hinkin 1976) do little to help IPM. Chabert and Blot (1992) suggest one wireworm/trap as a threshold for early planted maize based on their observations in northern France. Their work, however, does not discriminate the larvae captured and provides no statistics. From a practical point of view, the prevalent *A.* species in fields intended for maize crops need to be identified if the correct IPM thresholds are to be established. This could be achieved by: (a) a quick binocular observation of representative larvae samples collected from fields (this needs trained people; currently a trained technician can identify about 40 larvae/h); (b) PCR-based identification (Ellis et al. 2009; Staudacher et al. 2010); and (c) indirectly evaluating: (i) information from click beetle monitoring with pheromone traps (Furlan et al. 2001a; Furlan and Tóth 2007; Tóth et al. 2003) since captured click beetles may be correlated with the presence in the soil of same-species larvae, at least for the three main species considered herein (Furlan et al. 2001b) while this is uncertain for other important European species, such as *A. obscurus* L., *A. lineatus* L. and *A. sputator* L. (Benefer et al. 2012; Blackshaw and Hicks 2013; Landl et al. 2010); and (ii) the characteristics of the field (Blackshaw and Hicks 2013; Furlan et al. 2011; Hermann et al. 2013; Staudacher et al. 2013). From a practical point of view, when a restricted area is monitored, the main *Agriotes* species can be easily determined because the number of the main species is limited, and a trained IPM technician can therefore identify the larvae of the few species present based on their few discriminating characteristics. Furthermore, when field information (e.g. rotation, click beetle captures) is collected and mapped properly, technicians will only need to determine a few larvae to garner reliable information about the species involved, as the species in a field tends to remain the same for at least about 4–5 years if conditions remain unchanged (Furlan, personal observation). Further studies on the agronomic factors influencing crop response to wireworm damage (e.g. hybrids compensating for stand reduction) may improve the correlation between wireworm density and maize damage, as well as provide accurate (probably higher) thresholds for other groups of hybrids and for a range of conditions (e.g. irrigated or non-irrigated fields).

## Conclusion

The information herein may be used immediately to implement IPM and to tackle soil pests attacking maize in many European regions. As a result, it may lead to a considerable reduction in the use of soil pesticides and in a fall in the environmental impact of agriculture without negative repercussions on farmers’ income. This can be achieved with the procedure described in Furlan (2005): (i) locate the areas with a serious risk of wireworm attacks by assessing field/environmental factors (Hermann et al. 2013; Furlan and Talon 1997; Furlan et al. 2011; Staudacher et al. 2013); (ii) in areas at risk of wireworm attacks, assess current *Agriotes* populations with the aforementioned procedure, i.e. use bait traps and assess the actual average larval population, in fields intended for maize sowing; (iii) if the average number of wireworms does not exceed the thresholds established, maize may be sown without any treatment; if the average number of wireworms does exceed at least one of the thresholds, farmers have the option of moving maize to a no-risk field, as well as of applying organic treatments (Furlan 2007; Furlan et al. 2009b, 2010), or chemical treatments (Furlan et al. 2007, 2011 and Ferro and Furlan 2012). The aforementioned procedure may be considered the first reliable practical contribution towards implementing IPM of wireworms in Europe in accordance with EU Directive 2009/128/EC.
